# Hepatoprotective agents in the management of intrahepatic cholestasis of pregnancy: current knowledge and prospects

**DOI:** 10.3389/fphar.2023.1218432

**Published:** 2023-08-31

**Authors:** Dan Shan, Siyu Dai, Qian Chen, Yupei Xie, Yayi Hu

**Affiliations:** ^1^ Department of Obstetrics and Gynecology, West China Second University Hospital, Sichuan University, Chengdu, China; ^2^ Key Laboratory of Birth Defects and Related Diseases of Women and Children, Ministry of Education, Sichuan University, Chengdu, China

**Keywords:** hepatoprotective agents, intrahepatic cholestasis of pregnancy, liver function, pregnancy, ursodeoxycholic acid

## Abstract

Intrahepatic cholestasis of pregnancy (ICP) is characterized by unexplained distressing pruritus in the mother and poses significant risk to the fetus of perinatal mortality. Occurring in the second and third trimester, the serum bile acid and aminotransferase are usually elevated in ICP patients. Ursodeoxycholic acid (UDCA) is the first line drug for ICP but the effectiveness for hepatoprotection is to a certain extent. In ICP patients with severe liver damage, combination use of hepatoprotective agents with UDCA is not uncommon. Herein, we reviewed the current clinical evidence on application of hepatoprotective agents in ICP patients. The underlying physiological mechanisms and their therapeutic effect in clinical practice are summarized. The basic pharmacologic functions of these hepatoprotective medications include detoxification, anti-inflammation, antioxidation and hepatocyte membrane protection. These hepatoprotective agents have versatile therapeutic effects including anti-inflammation, antioxidative stress, elimination of free radicals, anti-steatohepatitis, anti-fibrosis and anti-cirrhosis. They are widely used in hepatitis, non-alcoholic fatty liver disease, drug induced liver injury and cholestasis. Evidence from limited clinical data in ICP patients demonstrate reliable effectiveness and safety of these medications. Currently there is still no consensus on the application of hepatoprotective agents in ICP pregnancies. Dynamic monitoring of liver biochemical parameters and fetal condition is still the key recommendation in the management of ICP pregnancies.

## Introduction

Intrahepatic cholestasis of pregnancy (ICP) occurs in the second and third trimesters of pregnancy. It is a rare disorder of unknown etiology. The prevalence of ICP varies from 0.1% to 15.6% and has significant geographical and demographic differences (Williamson and Geenes, 2014; Wood et al., 2018; Piechota and Jelski, 2020; Smith and Rood, 2020). ICP poses significant risk to the fetus of perinatal mortality. The manifestation in mothers is characterized by unexplained distressing pruritus with the elevation of bile acid. In ICP pregnancies, the hepatic transaminases are usually elevated due to the toxic effects of bile acid. Despite the fact that the severity of ICP is mainly based on the serum level of bile acid, significant increase in transaminases usually demonstrate extensive injury to hepatocyte and severe damage to liver function. Of note, the liver function is also closely associated with fetal conditions irrespective of the serum bile acid level. In pregnancies with elevated liver enzymes, the pregnancy outcome is not optimistic even when the bile acid is in an acceptable level (Bicocca et al., 2018; Azzaroli et al., 2020; Katarey and Westbrook, 2020; Lao, 2020; Pavelic et al., 2022).

As the only first-line drug approved by the U.S. Food and Drug Administration (FDA) for the treatment of cholestasis, Ursodeoxycholic acid (UDCA) is recommended in the treatment for ICP in several national guidelines (European-Association-for-the-Study-of-the-Liver, 2009; Obstetrics-Group-of-Obstetrics-and-Gynecology-Branch-of-Chinese-Medical-Association, 2015; Lee et al., 2021; Royal-College-of-Obstetricians-and-Gynaecologists, 2022). UDCA is a natural hydrophilic bile acid, the key mechanism of UDCA is to decrease the cholesterol saturation index of bile and suppress the absorption of cholesterol in intestine. UDCA is also proved to have the function of anti-inflammation, antioxidative stress, elimination of free radicals, regulation of immune balance and regulation of autophagy and apoptosis (Cabreraet al., 2019; Li et al., 2021; Shan et al., 2021). In the past several decades, the therapeutic value and safety of UDCA have been fully confirmed for alleviating symptoms in patients with ICP. But with the extensive use of UDCA, the limitation of this medication is also exposed. The effectiveness of UDCA is in a certain extent, especially in patients with severe liver damage (Shah and Kowdley, 2020). In this condition, hepatoprotective drugs should be applied. At present, the combination use of UDAC with hepatoprotective agents is often applied for treating ICP in clinical practice. Studies on these medications demonstrated effectiveness in hepatoprotection and with few adverse events. However, there is still no consensus on hepatoprotection treatment strategy for ICP patients. Based on the urgent need in clinical practice, this study aims to review the effectiveness and application of hepatoprotective drugs in the management of ICP patients.

## Hepatoprotective agents in the management of ICP

### S-adenosyl-L-methionine (SAMe)

S-Adenosyl-L-methionine (SAMe) is a molecule of significant importance for cell survival and function in all living organisms. It is recognized as the principal methyl donor reagent for methylation reactions. Considering the critical role of methylation in sustaining the normal cell function and maintaining the various cellular processes, any alterations in SAMe metabolisms can bring profound influence in cell growth, differentiation and normal function. SAMe is also the precursor of glutathione (GSH) (Lu, 2000). Since the structure been first unveiled in 1951 (Cantoni, 1951), studies investigating the biological function of SAMe have not been stopped. SAMe participate in tremendous physiological process and is recognized as the most important cofactor only second to adenosine triphosphate (ATP).

SAMe is involved mainly in three biological pathways: trans-methylation, trans-sulfuration and polyamine synthesis (Lu, 2000; Ouyang et al., 2020). More than 90% of the formed SAMe molecules are consumed to sustain methylation reactions (Ouyang et al., 2020). Liver is the major site for SAMe synthesis and degradation and have the fundamental role in the homeostasis of SAMe. In patients with liver disease, the intracellular SAMe is inadequately produced. But this inadequacy can be compensated from exogenous SAMe administration. Due to its crucial role in physiological processes and beneficial effect for hepatocyte, SAMe is widely used in liver disease including hepatitis, non-alcoholic fatty liver disease (NAFLD) and cholestasis. Its therapeutic effect is also proved in patients with neurological diseases, arthritis and cancer. Guidelines from Society for maternal and fetal medicine, European Association for the Study of the Liver and Chinese Medical Association all recommend the application of SAMe in the management of ICP(European-Association-for-the-Study-of-the-Liver, 2009; Obstetrics-Group-of-Obstetrics-and-Gynecology-Branch-of-Chinese-Medical-Association, 2015; Royal-College-of-Obstetricians-and-Gynaecologists, 2022). SAMe is also frequently applied in other types of cholestatic disease, including neonatal cholestasis and cholestasis caused by NAFLD (Virukalpattigopalratnam et al., 2013; Zeng et al., 2021). Evidence from both animal models and clinical trials revealed that exogenous administration of SAMe raised the GSH levels and ameliorated liver injuries (Mora et al., 2018; Brzački et al., 2019). In spite of its widely accepted therapeutic effects, the exact kernel mechanism of its function remains to be explored since SAMe is involved in so many critical cellular processes. But there is no doubt that the intracellular trans-methylation and trans-sulfuration processes are modulated due to the supplementation of exogenous SAMe.

Studies regarding the hepatoprotective effects of SAMe are summarized in (Supplementary Tables S1, S2) (Frezza et al., 1984; Frezza et al., 1990; Ribalta et al., 1991; Nicastri et al., 1998; Binder et al., 2006; Wang and Shen, 2012; Zhang et al., 2015; Jiang et al., 2019; Li and Xiao, 2019; Fanying et al., 2023). In this table, studies on the comparison between SAMe with placebo and SAMe in combination with UDCA are presented. Considering the first line medication for ICP is still UDCA, we did not analyze studies comparing the treatment effects between SAMe with UDCA. From these studies, we can see that in recent years, SAMe is usually applied with the combination of UDCA and seldomly used alone as the monotherapy for ICP patients. Compared with placebo, SAMe is found to be effective in reducing liver transaminases in studies conducted in the 1980s–1990s. Results from the combination use of SAMe plus UDCA indicated the combination therapy provided better maternal and fetal prognosis. Results from meta-analyses summarizing the treatment of ICP in recent decade demonstrated that UDCA-SAMe combination therapy is considered to have better hepatoprotective effects than either UDCA or SAMe monotherapy (Zhou et al., 2014; Zhang et al., 2016).

### Polyene phosphatidylcholine (PPC)

Polyene phosphatidylcholine (PPC), a major bioactive ingredient of essential phospholipids, have fundamental roles in maintaining the hepatocyte membrane fluidity and function (Li et al., 2021). The major component of PPC is phosphatidylcholine (PC). PC is the key component of organelle membranes and cell membrane. Polyunsaturated fatty acids such as linolenic acid and oleic acid are rich of PPC. By providing the endogenous phospholipids, the damaged liver cell and organelle membrane are repaired by PPC. The membrane function is restored and the fluidity and stability of cell membrane are increased, these mechanisms can protect hepatocyte from the variety of detrimental factors (Committee-of-the-treatment-with-polyenphosphatidylcholine-in-patients-with-liver-diseases, 2017). PPC is also proved to promote liver function restoration by recovering oxidative balance (Cao et al., 2016). Because of its function in repairing cell membranes and maintaining the integrity of biofilm. Recent studies suggest that PPC can be used as an anti-inflammatory drug (Pan et al., 2017; Feng et al., 2020).

PPC has been used vastly in the treatment for hepatitis, liver steatosis, NAFLD, alcoholic liver disease and drug-induced liver injuries. A multicenter retrospective study including 6,052 patients with various liver disease (8% Hepatitis B Virus (HBV) infected) demonstrated that PPC efficiently decreased alanine aminotransferase (ALT) and aspartate aminotransferase (AST) levels in patients with liver diseases regardless of the status of HBV infection. High-dose PPC resulted in a stronger effect than low-dose PPC(Xu et al., 2022). PPC has better hepatoprotective effect than glutathione or magnesium isoglycyrrhizinate even in patients with hepatic carcinoma (Li et al., 2022). In a prospective study including 2,843 adult patients with newly diagnosed NAFLD complicated with cardiometabolic comorbidities, PPC is found to improve the ultrasonographic features of NAFLD in these patients (Maev et al., 2020).

Three clinical trials focusing PPC in ICP are presented in (Supplementary Tables S1, S2) (Li and Yan, 2014; Jie et al., 2017; Zhu et al., 2022). All of the three trials proved the beneficial effects of PPC, the serum transaminases in ICP pregnancies were decreased and PPC did not cause adverse events for the fetus. Animal studies reveal consistent findings. Karaman. et all used a rat model of biliary obstruction. They found supplementation with polyunsaturated phosphatidylcholine alleviated the severity of liver damage and fibrosis in biliary obstructed rats (Karaman et al., 2003). Improvement in bile lipid secretion might be another underlying mechanism for the beneficial effects of PPC for ICP. Research from murine models found that supplementation with phosphatidylcholines promote the bile lipid secretion. They found phosphatidylcholines were required in the bile secrete process to form vesicles enriched in cholesterol. The protective effect of phosphatidylcholines could possibly be originated from the activation of multidrug export pump 1 (MDR1), which protects the hepatocyte by returning hydrophobic toxic bile components to the bile (Muller and Jansen, 1998; Chanussot and Benkoël, 2003).

### Glutathione (GSH)

Glutathione (GSH) is a thiol-containing tripeptide consisting of L-glutamate, cysteine, and glycine. GSH and its related enzymes, such as glutathione S-transferase (GSH-St) and glutathione peroxidase (GPx) are important antioxidants of human body that arrest toxic electrophilic xenobiotics and eliminate free radicals (Wu et al., 2004; Lv et al., 2019; Lai et al., 2020). Reduced glutathione is the major form of glutathione and is predominately distributed in the cytosol of mammalian cells. GSH is involved in several cellular metabolic activities and play important roles in maintaining the biochemical balance and physiological function. In mitochondria, GSH is oxidized to glutathione disulfide by GPx. During the oxidation process of GSH to glutathione disulfide by GPx, H_2_O_2_ is reduced to H_2_O. This catalytic process promotes the conversion of toxic peroxides to nontoxic hydroxyl compounds. By decomposition of H_2_O_2_, the detrimental effect of the peroxide is eliminated, and the structure and function of cell membranes are maintained (Wu et al., 2004; Lv et al., 2019). GSH in the nucleus is important in the cell cycle (Markovic et al., 2007). In endoplasmic reticulum (ER), the GSH/glutathione disulfide ratio is in a very high level to keep a highly oxidizing environment, in the condition of which the normal function of ER can be maintained (Cao and Kaufman, 2014).

Considering the fact the liver is the main supporter of total body GSH turnover, which contribute to at least 90% of GSH inflow into the systemic circulation (Lauterburg et al., 1984). Liver dysfunction inevitably lead to the impairment of GSH synthesis, which in turn cause the aggravation of damage to liver due to the inability to scavenge toxic peroxides and free radicals. In cirrhotic patients, the endogenous GSH basal appearance rate have been reported to have a 50% reduction (Bianchi et al., 1997). Supplementation with GSH seems a reliable way to alleviate the reduced GSH level in these patients and improve the antioxidant capacities. Evidence from rat model indicated that administration of oral GSH significantly increased the hepatic GSH level in rats (Vina et al., 1989). However, controversial results were found in the treatment effect of GSH to liver disease. Lai et used oral GSH with vitamin B6 to 61 patients with liver cirrhosis, they found no improvement in liver Child–Turcotte–Pugh scores, the oxidative stress was not reduced (Lai et al., 2020). In an open label, single arm, multicenter study including 34 patients with NAFLD, supplementation of GSH for 4 months lead to significant reduction in serum ALT level. In addition, triglycerides, non-esterified fatty acids, and ferritin levels also decreased (Honda et al., 2017). Another study included 75 patients diagnosed with HBV infection, 25 of them used oral GSH, the effective rate of GSH was 72% (Wang et al., 2008).

As summarized in (Supplementary Tables S1, S2), two Chinese studies investigated the therapeutic function of GSH in ICP patients (Ping et al., 2017; Chundong and Bin, 2022). From this limited evidence, it seems that GSH is an effective and safe medication for ICP. Studies exploring the treatment value and safety of GSH in ICP are still needed.

### Bicyclol

The Chinese medicinal herbals have been used for centuries and are proved to have the therapeutic effect in various liver diseases. Bicyclol [4,4′-dimethoxy-5,6,5′,6′-bis(methylenedioxy)-2-hydroxymethyl-2′-methoxycarbonyl biphenyl], derived from traditional Chinese medicine Schisandra chinensis (Wuweizi), is an innovative hepatoprotective and anti-inflammatory medication in China. Since its approval by the Chinese Food and Drug Administration in 2004, endeavor to explore its efficacy for clinical application from clinical trials and effort to uncover the underpinning mechanisms from basic research have not been stopped. The therapeutic effects of bicyclol have been proved to be versatile from retrieved literature, including anti-inflammation, antioxidative stress, elimination of free radicals, anti-steatohepatitis, anti-fibrosis and prevention against nuclear DNA damage (Liu, 2009; Li et al., 2020; Zhao et al., 2021; Jia et al., 2022). Bicyclol is also proved to have the regulating function of gut microbiota and prevent ferroptosis (Zhao et al., 2021; Li et al., 2022; Zhao et al., 2022).

The therapeutic effects of bicyclol are investigated in patients with hepatitis, NAFLD and acute drug-induced liver injury. Results from two reviews including randomized controlled trials of Hepatitis B Virus (HBV) and Hepatitis C Virus (HCV) patients demonstrated beneficial effects of bicyclol (Wu et al., 2006; Yang et al., 2007). A phase IV clinical trial including more than 2000 HBV cases completed 6 months of bicyclol, the normalized rate of serum ALT exceeded 60%, and AST exceeded approximately 50%. In addition, the HBV replication can also be inhibited by bicyclol. After 6 months of treatment, the negative conversion rate of hepatitis B e antigen (HBeAg) was approximately 20%. The therapeutic efficacy was higher in patients at a younger age (Yao et al., 2005). Studies focusing on combination therapy effect of bicyclol and lamivudine revealed that bicyclol is better than lamivudine in reducing liver enzymes in the early stage of treatment (Huang et al., 2005). For patients with HCV, a phase IV clinical trial proved that bicyclol at dosage of 75–150 mg per day for 6 months could reduce the ALT and AST normalized rate by 30%. The negative rate of HCV-RNA was reduced by approximately 20% (Yao et al., 2005). Combined treatment of bicyclol with ribavirin in patients with HCV also showed better efficacy for inhibiting the hepatocirrhosis (Sun et al., 2007). For patients with NAFLD, bicyclol was evaluated in a systematic review included twelve randomized controlled trials (RCTs) involving 1,008 patients (Li et al., 2020). Evidence from this study presents the liver function is significantly improved in either bicyclol monotherapy or combination therapy with another hepatoprotective agent. For patients with acute drug-induced liver injury, evidence from a multicenter randomized trail including 241 patients indicated the therapeutic effect of bicyclol appeared to be convincing without adverse effects, and bicyclol at a higher dosage (150 mg per day) showed higher efficacy (Tang et al., 2022).

To date, there are no clinical trials focusing the application of bicyclol in cholestatic patients. Investigated the bicyclol effects in a cholestatic mouse model (Zhao et al., 2021). They found bicyclol could mitigate liver damage in cholestatic mice by amplifying the levels of hydrophilic bile acid and improve liver histopathological indexes. Bicyclol promoted activation of autophagy by increasing the lipidation of LC3. Their results indicate that bicyclol is a promising therapeutic strategy for cholestasis. The underlying mechanism is possibly through regulation of the autophagy-mediated HMGB1/p62/Nrf2 pathway. Yang found bicyclol could decrease the hepatic transaminases and alleviate the fibrosis index in the rat liver (Yang, 2020). They concluded the alleviating effects of bicyclol for cholestasis was associated with the inhibition of cholesterol 7a-hydroxylase (CYP7A1), which is the rate-limiting enzyme for synthesis of bile acid. As a novel medication derived from traditional Chinese medicine, the underlying mechanism for bicyclol is still elusive. Researches aiming at revealing the mechanisms of bicyclol is still emerging. Clinical trials on the application of bicyclol in cholestatic patients, especially ICP, are urgently needed to pinpoint the potential beneficial effect in this population.

### Obeticholic acid (OCA)

Obeticholic acid (OCA) is a synthetic bile acid derivative and act as a potent agonist of farnesoid X receptor (FXR) (Pellicciari et al., 2002; Kjærgaard et al., 2021). It is the approved medication by the FDA and European Medicines Agency (EMA) for the treatment of patients with primary biliary cholangitis with inadequate response to UDCA (Beuers et al., 2015; Hirschfield et al., 2015; Nevens et al., 2016; Kjærgaard et al., 2021). In 17α-ethynyl estradiol induced ICP mouse model, OCA was found to activate placental, maternal, and fetal hepatic FXR signaling. The glutathione depletion and lipid peroxidation in placenta and fetal liver were attenuated with OCA, the placental protein nitration process was also suppressed. OCA almost completely suppressed the elevation of serum bile acid, ALT, and AST levels and markedly attenuated the necrosis and cytoplasm rarefaction of hepatocytes. The incidence of intrauterine growth restriction in the offspring was decreased by OCA supplementation (Chen et al., 2019). In C57BL/6J pregnant mouse model fed with cholic acid-enriched diet and OCA plus cholic acid enriched diet, OCA supplementation during gestation was found to have no apparent detrimental impact on maternal and fetal morphometry. The fetal hypercholanemia was greatly ameliorated with OCA (Pataia et al., 2020).

Currently, there are no clinical trials on OCA’s therapeutic effect in ICP patients. But the efficacy of OCA has been testified in patients with primary biliary cholangitis and non-alcoholic steatohepatitis and primary biliary cholangitis (Nevens et al., 2016; Kjærgaard et al., 2021; Kulkarni et al., 2021). In a 12-month, double-blind, placebo-controlled, phase 3 trial, 216 primary biliary cholangitis patients were randomized to OCA 10 mg, OCA 5–10 mg and placebo group. Patients in the two OCA groups had greater decreases than those in the placebo group in the ALT level and total bilirubin level (Nevens et al., 2016). In a meta-analysis including seven RCTs of 2,834 patients with non-alcoholic steatohepatitis and primary biliary cholangitis, OCA was found to improve the hepatic fibrosis. But OCA at higher dosage (25 mg) was also associated with higher incidence of pruritus and discontinuation of the treatment (Kulkarni et al., 2021). The pros and cons of OCA in ICP patients should be investigated in future research.

### Traditional Chinese medicine (TCM)

Traditional Chinese medicine (TCM) have been widely used in the treatment of hepatic diseases including NAFLD, hepatitis, cirrhosis, and cholestasis in Asia for centuries. The treatment concept of TCM for hepatic disease is holistic. Advantage from this is a more individualized treatment plan. In China, TCM are widely applied with combination of UDCA for the treatment of ICP (Jiang et al., 2021; Wei et al., 2022). The treatment for ICP mainly focused hepatoprotection and promoting circulation of bile. The frequently used herbs for ICP included Yinchenhao decoction, Lidan Yishen Decoction, Jinying Huayu Decoction, Yin Huang Mixture, Dan Shen (Radix Salvia Miltiorrhiza) etc. The various TCM used could manifest with multiple features and functions in mechanism, including anti-oxidant stress, regulation of lipid metabolism, anti-inflammation, anti-fibrosis and modulation of gut microbiota (Liang et al., 2020; Dai et al., 2021; Wei et al., 2022).

Yinchenhao decoction is a classic TCM for cholestasis. This decoction consists of Artemisia capillaris Thunb, Gardenia, and Rhubarb. Artemisia capillaris Thunb is the most important medicinal material, which is named as Yinchenhao in China. This medicinal plant has over 100 kinds of active chemical components such as coumarin compounds flavonoids and organic acids (Zhang et al., 2013; Huang et al., 2021; He et al., 2023; Yao et al., 2023). The pharmacological activities of Yinchenhao includes anti-inflammatory, anti-fibrosis, and anti-tumor effects (Zhang et al., 2013; Li, 2020). Yinchenhao decoction could downregulate transforming growth factor β1 (TGF-β1), p-Smad3 and extracellular regulated protein kinases 1/2 (ERK1/2) expression in chenodeoxycholic acid activated hepatic stellate cells and increase the cell viability significantly (Cai et al., 2018). The most abundant compounds in Yinchenhao decoction were 7-methoxycoumarin, chrysophanol, deoxychrysoside-8-O-gallic acid salt, emodin, and mussaenosidic acid, which can be detected in the plasma of patients taking Yinchenhao decoction (Yao et al., 2023). With the ability of downregulation the level of hepatocyte apoptosis and alleviate oxidative stress, 7-methoxycoumarin can protect liver injury induced by carbon tetrachloride (CCl4) (Sancheti et al., 2013). Chrysophanol can alleviate the liver fibrosis progress by regulating GPx function and endoplasmic reticulum stress response (Kuo et al., 2020). Mussaenosidic acid can regulate immune function by inhibiting the classical complement pathway, and has antioxidant activity in Fe2+-Cystine induced rat liver damage model (Zhang et al., 2010). Emodin can play hepatoprotective effect by acting as the regulating factor for oxidative stress and autophagy (Zheng et al., 2019; Lee et al., 2020). A network meta-analysis by included 38 RCTs exploring the effect of Yinchenhao in ICP pregnancies. Their results demonstrated that when compared with UDCA used alone, Yinchenhao decoction plus UDCA dramatically reduced the serum levels of bile acid, ALT, and AST (Jiang et al., 2021). Recent clinical trials of Yinchenhao in ICP pregnancies are summarized in Supplementary Tables S1, S2 (Wang et al., 2019; Chen et al., 2020; Xu et al., 2021; Zhu, 2021) (Supplementary Tables S1, S2). Based on the hepatoprotective effects of Artemisia capillaris Thunb, the Yin Huang Mixture is another clinical experiential decoction with the combination of different herbs including *Hypericum japonicum* Thunb, Eucommia ulmoides Oliver, Rheum officinale Baill, Gardenia jasminoides Ellis, Poria cocos Wolf and Dictamnus dasycarpus Turcz. Animal studies using cholestatic rat model found that the Yin Huang Mixture can reduce bile acid level by upregulating the hepatobiliary transporters multidrug resistance associated protein 2 (MRP2) and bile salt export pump (BSEP) (Liu, Hou, and Zhao, 2018).

### Other medications with hepatoprotective effect

#### Silymarin

Silymarin is the extract of Silybum marianum and consists of seven flavonolignans and a flavonoid (Federico, Dallio, and Loguercio, 2017). The major active compound is silybin, which account for 70% of the total composition of silymarin and has a remarkable biological effect. Silymarin has long been used in various liver disorders, including hepatitis, NAFLD, cirrhosis and hepatocellular carcinoma. In spite of its antioxidant and anti-inflammatory effect, silymarin has direct antiviral effect. Silymarin is found to be able to intervene in multiple therapeutic targets of liver diseases, the fat accumulation process in liver, hepatocyte mitochondria function and even insulin resistance are modulated with silymarin treatment (Federico et al., 2017; Gillessen and Schmidt, 2020). Studies on application of silymarin for cholestatic patients are limited. One case report study reported silymarin usage in ICP patients. The patient’s liver enzymes recovered after 4 weeks of silymarin treatment and no adverse pregnancy outcomes were observed (Han et al., 2017).

#### CoQ10

The therapeutic value of Co Q10 for ICP pregnancy is from animal studies. As an endogenous redox-active substance essential for mitochondrial respiratory chain, Co Q10 is considered to be the primary regenerating antioxidant and have essential role against oxidative damage (Barshop and Gangoiti, 2007). In ICP patients, CoQ was found in a lower level than heathy pregnancies (Martinefski et al., 2014; Martinefski et al., 2020a). This indicated the overload of peroxide damage in ICP patients because Co Q10 is considered to be an early marker for oxidative stress (Martinefski et al., 2014). In cholestatic rat model, combined administration of CoQ10 and UDCA reduced the bile acid and transaminases level and also prevented the fall in blood glutathione, indicating a more favorable redox environment in the liver (Martinefski et al., 2020b).

## Discussion

The successful management for ICP includes bidirectional goals. Endeavors should be made to prevent fetal complications and efforts on reducing the maternal clinical symptoms and normalizing biochemical indicators should also be paid. As the first line drug for ICP, UDCA is recommended in several national guidelines. In spite of the anticholestatic function, UDCA is also proved to play hepatoprotective roles (Walker et al., 2020). However, benefits from monotherapy of UDCA are far from adequate. In patients with severe liver injury, hepatoprotective medications must be applied to prevent further damage in the liver.

In an uncomplicated pregnancy, the biosynthesis and detoxification processes are enhanced in the maternal liver to sustain the increased physiological needs during pregnancy. The hormonal, biochemical, and hematological changes in maternal liver mimic the physiopathological changes of chronic liver disease (Katarey and Westbrook, 2020). In ICP pregnancies, this change deteriorates. The nuclear factor-k-gene binding (NF-kB)-mediated proinflammatory cytokine production is activated due to the high concentration of cytotoxic bile acids (Chiang and Ferrell, 2018). Toxic bile acids damage the bile duct epithelium, and the enterohepatic circulation of bile acid is disrupted. With the accumulation of bile acid, the bile duct is ruptured and expose hepatocytes directly to high concentrations of bile acids. The direct toxic effects and following enhanced inflammatory reactions caused by excessive bile acid could lead to hepatocyte cell death and damaged liver function.

By promoting the regeneration of hepatocyte or enhancing detoxification, hepatoprotective drugs refer to the kind of medications that can improve liver function. Literally there is no consensus in regard to its classification. The basic pharmacologic functions of these medications include detoxification, anti-inflammation, antioxidation and hepatocyte membrane protection (Li et al., 2021). Information from the recommendations on hepatoprotective medications for ICP from the guidelines is very limited. Society for Maternal-Fetal Medicine suggested application of SAMe and cholestyramine as alternative drugs in patients cannot take UDCA (Lee et al., 2021). When dealing with ICP patients with significantly elevated hepatic aminotransferases, it is not uncommon for obstetricians to choose the combination use of one or more hepatoprotective drugs with UDCA for ICP treatment in clinical practice. The common combination is usually SAMe and UDCA, but despite the lack of adequate evidence-based medical evidence, addition of the second hepatoprotective drug is mainly based on the doctors’ clinical experience and preference. Factors including patient’s personal characteristics, level of liver enzymes and bile acid, and compliance of the patient should be taken into consideration when proscribing these hepatoprotective agents. Suggestions from gastroenterologist should be adopted in ICP patients with severe liver damage.

Of note, there is growing evidence suggesting the association of ICP with elevated risks for developing preeclampsia, gestational diabetes, and metabolic disorders (Martineau et al., 2015; Shan et al., 2016; Menżyk et al., 2018; Valdovinos-Bello et al., 2023). The altered glucose and lipid metabolism of ICP patients could be partially contributed by the reduction in the activity of FXR and Takeda G protein-coupled receptor (TGR5). (Ma et al., 2006; Renga et al., 2010; Seyer et al., 2013; Menżyk et al., 2018). With the reduction in the FXR activity, gluconeogenesis process and secretion function of β cell were influenced, leading to the impaired glucose tolerance observed in ICP. Enteric bile acid could stimulate TGR5 and result in further dysregulated synthesis of insulin and glucagon (Roberts et al., 2011; Parker et al., 2012). The impairments of FXR and TGR5 were also involved in the dysregulated synthesis and metabolism of lipid profiles in ICP pregnancies (de Aguiar Vallim et al., 2013; Chiang and Ferrell, 2020; Zhan et al., 2022). In ICP patients complicated with obesity, risks for development of PE, GDM and other hepatopathies were even further increased, suggesting a more severe dysregulation in the metabolic homeostasis (Valdovinos-Bello et al., 2023). However, recent studies exploring the hepatoprotective treatments in ICP were focused on the therapeutic effect in hepatic biochemical indicators, the improvement in the comorbidities of ICP have not been investigated. Recent protocols have been published aiming to explore UDCA’s efficacy in regulation of glucose level in GDM patients (Lovell et al., 2022). But evidence regarding UDCA’s beneficial effect in the alleviation of ICP related maternal metabolic disorders has not been explored. Similar with UDCA, hepatoprotective agents could also be identified as a potential promising treatment choice for the improvement of ICP related comorbidities. But whether alleviation in damaged liver function could have beneficial effect in reducing the related comorbidities is still unknown. As the underlying pathophysiological mechanism of ICP remains a mystery, whether the dysregulated glucose and lipid homeostasis coexist with ICP is unexplained. However, considering the fundamental role of liver as a metabolizing organ, improvement in liver function could no doubt promote better glucose and lipid metabolism and maintain the homeostasis of internal environment in ICP pregnancies. The therapeutic value of these medications in alleviating the risk for metabolic disorders in ICP patients should be further investigated.

**FIGURE 1:**
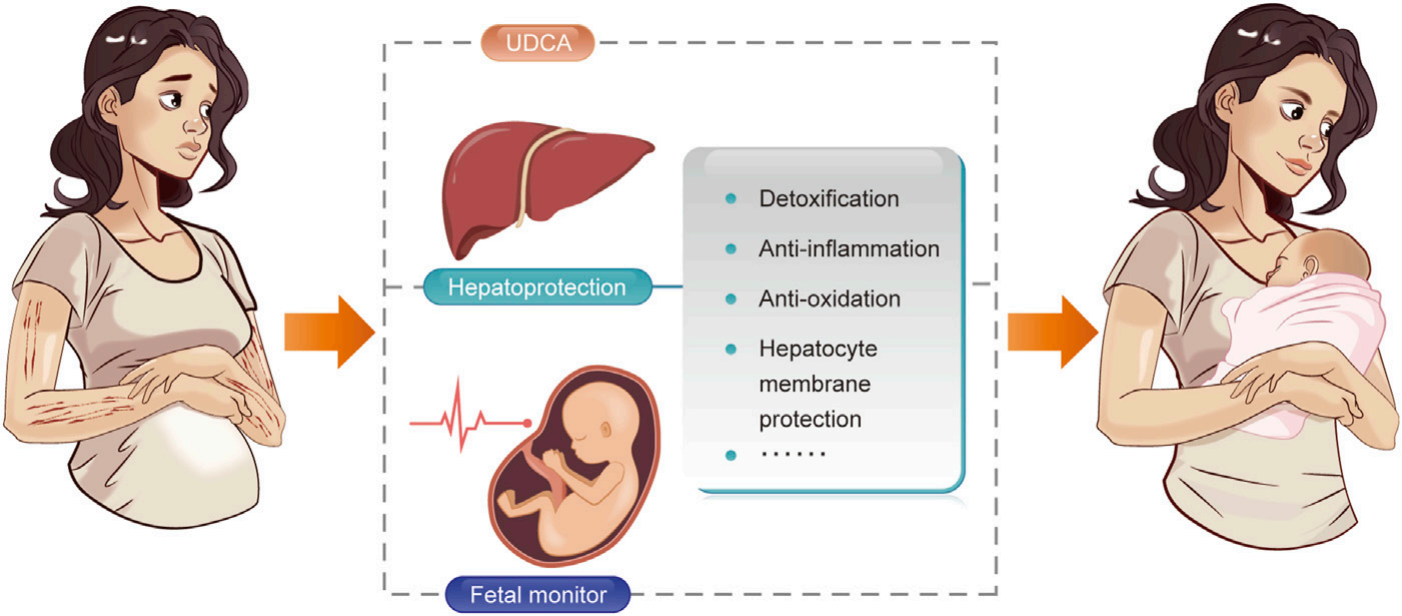
Management of patients with intrahepatic cholestasis of pregnancy.

The present study summarized the commonly used medications for hepatoprotection in ICP. In spite of UDCA and the hepatoprotective agents, regular monitoring of maternal and fetal conditions still has a critical position in the management of ICP (Figure 1). Our findings indicate the hepatoprotective effectiveness of the above-mentioned medications. Severe adverse events associated with these medications have not been identified in ICP patients. However, we should still bear in mind that evidence of some of the medications was from limited data, the safety parameters are still under investigation. Combination treatment of these hepatoprotective agents should be with caution considering the possibility of drug induced liver injury and lack of evidence from high quality studies. Future prospective studies with large sample size investigating the efficacy and safety of these hepatoprotective agents are needed. The therapeutic effect in improving ICP related comorbidities also needs to be explored.

## Conclusion

Although the prevalence of ICP is in low level, the potential detrimental outcomes of this pregnancy related complication call for more concern. The first line treatment for ICP is UDCA. In patients with severe liver damage, choosing an effective hepatoprotective treatment plan for ICP patients is of great significance. Factors including patient's personal characteristics, level of liver enzymes and bile acid, and compliance of the patient should be taken into consideration. Suggestions from gastroenterologist should be adopted. Limitations of this review included the limited evidence of the therapeutic effect of some medications in ICP patients. The quantitative analysis of the efficacy of these drugs was not performed either. Similar with UDCA, hepatoprotective agents could also be recognized as the treatment with promising effects in alleviating ICP related comorbidities. The efficacy and safety of these hepatoprotective medications need further exploration, and combination treatment should be with caution. In addition to UDCA and hepatoprotective agents, dynamic monitoring of maternal and fetal condition is still of great significance.

## Figures and Tables

**Table:** 

**ALT**	Alanine aminotransferase
**AST**	Aspartate aminotransferase
**ATP**	Adenosine triphosphate
**BSEP**	Bile salt export pump
**CCl4**	Carbon tetrachloride
**CYP7A1**	Cholesterol 7a-hydroxylase
**EMA**	Endoplasmic reticulum
**ERK1/2**	Extracellular regulated protein kinases 1/2
**FDA**	Food and Drug Administration
**FXR**	Farnesoid X receptor
**GPx**	Glutathione peroxidase
**GSH**	Glutathione
**GSH-St**	Glutathione S-transferase
**HBV**	Hepatitis B Virus
**HBeAb**	Hepatitis B e antibody
**HBeAg**	Hepatitis B e antigen
**HCV**	Hepatitis C Virus
**HMGB1**	High-mobility group box 1
**ICP**	Intrahepatic cholestasis of pregnancy
**LC3**	Microtubule-associated protein 1 light chain 3
**MDR1**	Multidrug export pump 1
**MRP2**	Multidrug resistance associated protein 2
**NAFLD**	Non-alcoholic fatty liver disease
**NF-kB**	The nuclear factor-k-gene binding factor
**Nrf2**	E2-related factor 2
**OCA**	Obeticholic acid
**PC**	Phosphatidylcholine
**PPC**	Polyene phosphatidylcholine
**RCT**	Randomized controlled trials
**SAMe**	S-Adenosyl-L-methionine
**TCM**	Traditional Chinese medicine
**TGF-β1**	Transforming growth factor β1
**UDCA**	Ursodeoxycholic acid
