# Supplementary material for: Hepatoprotective agents in the management of intrahepatic cholestasis of pregnancy: current knowledge and prospects
Source: Front Pharmacol. 2023 Aug 31;14:1218432. doi: 10.3389/fphar.2023.1218432 (PMC10500604; doi:10.3389/fphar.2023.1218432)
Supplement: Supplementary file 1 [file Table1.docx]

# Supplementary Table 1. Hepatoprotective agents in the management of ICP patients.

| **Author** | **Year** | **Country** | **Type of Study** | **Number of participants and application of medication** | **Outcome (in study group）** | **For Neonatal** |
| --- | --- | --- | --- | --- | --- | --- |
| **S-adenosylmethionine（SAMe）** | | | | | | |
| Binder | 2006 | ﻿Czech Republic | RCT | Study group 1: SAMe (1000mg IV per day and 1000mg oral per day, total duration 3 weeks), 25 ICP patients.  Study group 2: UDCA, 26 ICP patients.  Study group 3: UDCA + SAMe (1000mg IV per day and 1000mg oral per day, total duration 3 weeks), 27 ICP patients. | No difference between group 2 and group 3 in serum transaminases (ALT, AST，ALP) and TBA level. No difference in TBIL level. | No difference between group 2 and 3 in fetal distress rate, MASF rate and preterm rate. |
| Frezza | 1984 | Italy | RCT | Study group: SAMe (200 mg IV per day for 20 days), 6 ICP patients. SAMe (800 mg IV per day for 20 days), 6 ICP patients.  Control group: Placebo, 6 ICP patients. | Serum γGT, DBIL and TBA were lower in the study group. Pruritus score was lower in study group (high dosage). | No relevant results were reported. |
| Frezza | 1990 | Italy | RCT | Study group: SAMe (800 mg IV per day for 18 days), 15 ICP patients.  Control group: Placebo, 15 ICP patients. | Serum transaminases, TBIL and TBA were lower in study group. | The preterm labor rate was lower. |
| Jiang | 2019 | China | RCT | Study group 1: UDCA + SAMe (1000mg IV per day for 3 weeks), 38 ICP patients.  Control group: UDCA, 38 ICP patients. | Serum transaminases (ALT, AST), TBA and TBIL were lower in study group. Pruritus score was lower in study group. | The fetal distress rate and MASF rate were lower in study group. |
| Li | 2019 | China | Retrospective | Study group 1: UDCA + SAMe (1000mg IV per day for 14 days), 51 ICP patients.  Control group: UDCA, 51 ICP patients. | Serum transaminases (ALT, AST), TBA, CG and TBIL were lower in study group.  Serum IL-17 was lower in study group and TGF-β was higher in study group. | The preterm labor rate and fetal distress rate were lower. |
| ﻿Nicastri | 1998 | Italy | RCT | Study group 1: UDCA for 20 days, 8 ICP patients.  Study group 2: SAMe (800 mg IV per day for 20 days), 8 ICP patients.  Study group 3: UDCA + SAMe for 20 days, 8 ICP patients.  Control: Placebo, 8 ICP patients. | The reduction in TBIL, bile salts and pruritus score were improved in group 3 compared with group 1. | No difference in preterm labor rate and fetal distress rate. |
| Ribalta | 1991 | Chile | RCT | Study group: SAMe (800 mg IV per day for 20 days), 9 ICP patients.  Control group: Placebo, 9 ICP patients. | No difference in serum transaminases (ALT, AST) and TBA. | No difference in fetal distress rate. |
| Wang | 2012 | China | Retrospective | Study group 1: UDCA + GSH + SAMe (1000mg IV per day for 2 weeks), 112 ICP patients.  Control group: UDCA + GSH, 112 ICP patients. | Time to control pruritus was lower in study group. | The preterm labor rate and fetal distress rate were lower in study group. |
| Zeng | 2023 | China | Retrospective | Study group 1: UDCA + SAMe (1000mg IV per day for 3 weeks), 65 ICP patients.  Control group: UDCA, 65 ICP patients. | Serum transaminases (ALT, AST, ALP), TBA and CG were lower in study group. Time to control pruritus was lower in study group. | The preterm labor rate and fetal distress rate were lower in study group. The Apgar score at 1^st^ minute was higher in study group. |
| Zhang | 2015 | China | Multicenter RCT | Study group 1: UDCA, 41 ICP patients.  Study group 2: SAMe (1000mg IV per day for 2 weeks), 38 ICP patients.  Study group 3: UDCA + SAMe (1000mg IV per day for 2 weeks), 41 ICP patients. | No difference in serum transaminases (ALT, AST), TBA and TBIL between group 1 and 3. | No difference in rates of preterm delivery and MASF between group 1 and 3. No difference of Apgar score at 1^st^ and 5^th^ minute between group 1 and 3. |
| **Polyene Phosphatidylcholine (PPC)** | | | | | | |
| Cao | 2017 | China | Retrospective study | Study group: UDCA + SAMe + PPC (697.5 mg IV per day for 2 weeks), 45 ICP patients.  Control group: UDCA + SAMe, 45 ICP patients. | Serum transaminases (ALT, AST) and TBA were lower in study group. Pruritus score was lower in study group. | The fetal distress rate and MASF rate were lower in study group. No difference in preterm rate. |
| Li | 2014 | China | RCT | Study group: UDCA + SAMe + PPC (697.5 mg IV per day for 10 days), 40 ICP patients.  Control group: UDCA + SAMe, 40 ICP patients. | Serum transaminases (ALT, AST) and TBA were lower in study group. No difference in pruritus score between groups. | The fetal distress rate, preterm rate and MASF rate were lower in study group. |
| Zhu | 2022 | China | RCT | Study group: UDCA + PPC (697.5 mg IV per day for 2 weeks), 40 ICP patients.  Control group: UDCA, 40 ICP patients. | Serum transaminases (ALT, AST), bilirubin (DBIL, TBIL) and TBA were lower in study group.  Cholinesterase and APOA1 were higher in study group.  Time for alleviation of jaundice and control pruritus was shorter in study group. | The MASF rate were lower in study group. No difference in fetal distress rate. |
| **Reduced glutathione (GSH)** | | | | | | |
| Wang | 2022 | China | RCT | Study group: SAMe + GSH (1.8 g IV per day for 4 weeks), 45 ICP patients.  Control group: SAMe, 45 ICP patients. | Serum transaminases (ALT, AST, ALP), bilirubin (DBIL, TBIL) and TBA were lower in study group.  SOD and GSH-Px were higher in study group.  MDA and LPO were lower in study group. | No difference in fetal distress rate, MASF rate and preterm rate. |
| Xie | 2017 | China | RCT | Study group: UDCA + SAMe + GSH (2.4g IV per day for 30 days), 59 ICP patients.  Control group: UDCA + SAMe, 58 ICP patients. | Serum transaminases (ALT, AST) and TBA were lower in study group. Pruritus score was lower in study group. | No difference in fetal distress rate and preterm rate and Apgar score at 1^st^ minute. |
| **Yinchenhao decoction** | | | | | | |
| Chen | 2020 | China | RCT | Study group: SAMe+Yinchenhao decoction （twice per day, for 14 days）, 30 ICP patients.  Control group: SAMe, 30 ICP patients. | Serum TBA was lower in study group. Serum IL-17 was lower in study group and TGF-β was higher in study group.  Pruritus score was lower in study group. | The fetal distress rate, preterm rate and MASF rate were lower in study group. |
| Wang | 2019 | China | RCT | Study group: UDCA+SAMe+Yinchenhao decoction （twice per day, for 14 days）, 75 ICP patients.  Control group: UDCA+SAMe, 75 ICP patients. | Serum transaminases (ALT, AST), bilirubin (DBIL, TBIL) and TBA were lower in study group.  Pruritus score was lower in study group. | The fetal distress rate and preterm rate were lower in study group. |
| Xu | 2021 | China | RCT | Study group: Yinchenhao decoction （three times per day, for 7 days）, 38 ICP patients.  Control group: UDCA, 40 ICP patients. | No difference in serum transaminases (ALT, AST) and TBA level between groups.  Pruritus score was lower in study group. | The MASF rate was lower in study group. No difference in Apgar score at 1^st^ minute. |
| Zhu | 2021 | China | RCT | Study group: UDCA+Yinchenhao decoction （once per day, for 14 days）, 15 ICP patients.  Control group: UDCA, 15 ICP patients. | Serum transaminases (ALT, AST) and TBA were lower in study group. | The fetal distress rate, preterm rate and MASF rate were lower in study group. |

**Abbreviations:**

ALT, alanine aminotransferase; ALP, alkaline phosphatase; AST, aspartate aminotransferase; APOA1, Apolipoprotein A1; DBIL, direct bilirubin; CG, courage acid; GPx, glutathione peroxidase; GSH, Reduced glutathione; γGT, γ-glutamyl transpeptidase; ICP, Intrahepatic cholestasis of pregnancy; IL-17, interleukin 17; IV, intravenous; LPO, lipid peroxide; MASF, meconium-staining amniotic fluid; MDA, malondialdehyde; PPC, Polyene phosphatidylcholine; RCT, randomized controlled trial; SAMe, S-adenosylmethionine; SOD, superoxide dismutase; TBIL, total bilirubin; TGF-β, transforming growth factor-β; UDCA, Ursodeoxycholic acid.
