# Supplementary material for: Hepatoprotective agents in the management of intrahepatic cholestasis of pregnancy: current knowledge and prospects
Source: Front Pharmacol. 2023 Aug 31;14:1218432. doi: 10.3389/fphar.2023.1218432 (PMC10500604; doi:10.3389/fphar.2023.1218432)
Supplement: Supplementary file 2 [file Table2.docx]

**Supplementary Table 2: Biochemical parameters and symptoms of ICP after treatment.**

|  |  | Study group | Control group | p |  | Study group | Control group | p |
| --- | --- | --- | --- | --- | --- | --- | --- | --- |
| **SAMe** | | | | | | | | |
| Binder 2006 |  |  |  |  |  |  |  |  |
|  | ALT(μkat/L) | 1.5±1.46 | 1.7±2.22 | >0.05 | AST(μkat/L) | 0.93±0.87 | 0.78±1.31 | >0.05 |
|  | ALP(μkat/L) | 3.6±0.84 | 3.9±0.78 | >0.05 | TBA(μmol/L) | 20±22.18 | 18±28.25 | >0.05 |
|  | TBIL(μmol/L) | 9.0±4.73 | 8.2±3.72 | >0.05 |  |  |  |  |
| Frezza 1984 |  |  |  |  |  |  |  |  |
|  | Pruritus score | 1.33± 0.5 | 3.0± 0.8 | <0.05 |  |  |  |  |
| Jiang 2019 |  |  |  |  |  |  |  |  |
|  | ALT(U/L) | 101.26± 30.25 | 143.26± 29.5 | 0.000 | AST(U/L) | 97.25±28.67 | 131.02± 32.58 | 0.000 |
|  | TBA(μmol/L) | 31.08±9.65 | 42.12± 10.36 | 0.000 | TBIL(μmol/L) | 18.54±5.48 | 22.62±6.43 | 0.004 |
|  | Pruritus score | 0.63±0.45 | 1.02±0.54 | 0.001 |  |  |  |  |
| Li 2019 |  |  |  |  |  |  |  |  |
|  | ALT(U/L) | 45.35± 9.38 | 70.36± 13.54 | <0.001 | AST(U/L) | 52.21± 9.31 | 72.64± 15.84 | <0.001 |
|  | TBA(μmol/L) | 13.24± 2.15 | 19.53± 4.49 | <0.001 | TBIL(μmol/L) | 15.21± 3.25 | 18.51± 4.07 | <0.001 |
|  | CG (mg/L) | 16.10± 3.56 | 21.02± 5.02 | <0.001 | IL-17 (ng/L) | 2.25± 0.56 | 3.49± 0.67 | <0.001 |
|  | TGF-β(ng/L) | 5.82± 1.24 | 3.89± 0.76 | <0.001 | Pruritus score | 1.22± 0.68 | 3.08± 1.08 | <0.001 |
| Nicastri 1998  (Change from base line) |  |  |  |  |  |  |  |  |
|  | ALT (U/L) | 149.9±34.0 | 131.1±19.3 | 0.2 | ALP(U/L) | 201.4±13.9 | 181.2±26.4 | 0.08 |
|  | TBIL(mg/dL) | 2.0±0.4 | 1.2±0.4 | 0.0021 | Bile salts (μmol/L) | 44.3±8.6 | 33.0±10.0 | 0.0296 |
|  | Pruritus score | 1.8±0.5 | 0.8±0.3 | 0.0003 |  |  |  |  |
| Wang 2012 |  |  |  |  |  |  |  |  |
|  | Time to control pruritus (day) | 3.6±1.4 | 6.2±2.2 | <0.05 |  |  |  |  |
| Zeng 2023 |  |  |  |  |  |  |  |  |
|  | ALT(U/L) | 48.63±9.42 | 72.95±10.33 | <0.001 | AST(U/L) | 54.97±10.05 | 75.09±17.82 | <0.001 |
|  | ALP(U/L) | 140.32±17.93 | 156.72±19.09 | <0.001 | TBA(μmol/L) | 12.75±2.17 | 20.24±3.92 | <0.001 |
|  | CG (mg/L) | 10.12±2.14 | 16.25±3.42 | <0.001 |  |  |  |  |
| Zhang 2015 |  |  |  |  |  |  |  |  |
|  | ALT(U/L) | 126.8±100.07 | 113.46±86.93 | 0.874 | AST(U/L) | 88.12±68.84 | 80.76±54.76 | 0.807 |
|  | TBA(μmol/L) | 21.64±17.2 | 18.66±16.9 | 0.478 | TBIL(μmol/L) | 13.74±4.9 | 12.7±6.89 | 0.859 |
| **PPC** | | | | | | | | |
| Cao 2017 |  |  |  |  |  |  |  |  |
|  | ALT(U/L) | 30.16±2.15 | 46.72±2.77 | <0.05 | AST(U/L) | 29.89±2.19 | 33.16±2.41 | <0.05 |
|  | TBA(μmol/L) | 12.38±0.25 | 20.51±0.62 | <0.05 | Pruritus score | 1.19±0.21 | 2.61±0.39 | <0.05 |
| Li 2014 |  |  |  |  |  |  |  |  |
|  | ALT(U/L) | 98.67±72.25 | 105.25±97.25 | <0.05 | AST(U/L) | 52.89±15.27 | 82.65±46.33 | <0.05 |
|  | TBA(μmol/L) | 13.06±10.32 | 20.03±16.50 | <0.05 | Pruritus score | 0.36±0.21 | 0.59±0.13 | <0.05 |
| Zhu 2022 |  |  |  |  |  |  |  |  |
|  | ALT(U/L) | 53.69±10.8 | 68.07±12.34 | <0.001 | AST(U/L) | 37.47±5.85 | 49.16±6.94 | <0.001 |
|  | TBA(μmol/L) | 12.43±2.84 | 15.67±3.13 | <0.001 | TBIL(μmol/L) | 7.49±1.12 | 12.64±1.36 | <0.001 |
|  | DBIL(μmol/L) | 5.33±0.98 | 8.49±1.21 | <0.001 | Cholinesterase(U/L) | 5939.41±1236.8 | 5368.68±1125.97 | 0.034 |
|  | APOA1(U/L) | 1.93±0.38 | 1.75±0.4 | 0.042 | Time to control pruritus (day) | 3.41±0.97 | 4.96±1.23 | < 0. 001 |
|  | Time to control jaundice (day) | 14.68±4.26 | 19.28±5.47 | <0.001 |  |  |  |  |
| **GSH** | | | | | | | | |
| Wang 2022 |  |  |  |  |  |  |  |  |
|  | ALT(U/L) | 85.67±11.65 | 131.48±15.7 | 0.00 | AST(U/L) | 109.63±10.79 | 158.63±12.6 | 0.00 |
|  | ALP(U/L) | 50.96±10.37 | 87.65±11.57 | 0.000 | TBA(μmol/L) | 18.62±3.78 | 29.67±5.27 | 0.000 |
|  | TBIL(μmol/L) | 18.65±3.13 | 24.71±3.29 | 0.000 | DBIL([μ](http://www.baidu.com/link?url=fKjYR9yH3i4D3caiFm5J7c78oCZT9rcK10GMakdfRK-rBqJsJdm1wFxVr_q2oL74kpakLCNEn3j6-9jHm1xmI_)mol/L) | 6.25±2.69 | 9.52±2.78 | 0.000 |
|  | SOD(mmol/L | 0.18±0.03 | 0.14±0.02 | 0.000 | MDA(ng/L) | 6.85±3.82 | 12.24±5.15 | 0.000 |
|  | GPx(mmol/L) | 42.12±8.23 | 31.56±7.54 | 0.000 | LPO(μmol/L) | 1.08±0.19 | 1.82±0.25 | 0.000 |
| Xie 2017 |  |  |  |  |  |  |  |  |
|  | ALT(U/L) | 94.06±47.25 | 122.83±51.76 | 0.025 | AST(U/L) | 51.16±22.95 | 78.50±28.86 | <0.001 |
|  | TBA(μmol/L) | 14.47±5.86 | 21.17±9.30 | <0.001 | Pruritus score | 0.42±0.17 | 0.78±0.20 | <0.001 |
| **Yinchenhao Decoction** | | | | | | | | |
| Chen 2020 |  |  |  |  |  |  |  |  |
|  | TBA(μmol/L) | 10.15±4.23 | 12.39±4.21 | 0.000 | IL-17 (ng/L) | 2.70±0.82 | 3.61±0.92 | 0.000 |
|  | TGF-β(ng/L) | 5.63±061 | 3.73±0.39 | 0.003 | Pruritus score | 1.02±0.59 | 1.51±0.71 | 0.007 |
| Wang 2019 |  |  |  |  |  |  |  |  |
|  | ALT(U/L) | 34.51±6.32 | 44.51±8.56 | <0.05 | AST(U/L) | 43.59±6.39 | 52.89±6.39 | <0.05 |
|  | TBA(μmol/L) | 15.50±1.39 | 25.50±3.12 | <0.05 | DBIL([μ](http://www.baidu.com/link?url=fKjYR9yH3i4D3caiFm5J7c78oCZT9rcK10GMakdfRK-rBqJsJdm1wFxVr_q2oL74kpakLCNEn3j6-9jHm1xmI_)mol/L) | 6.51±1.58 | 9.51±1.96 | <0.05 |
|  | TBIL(μmol/L) | 38.51±6.32 | 48.51±8.56 | <0.05 | Priritus score | 1.51±0.03 | 2.34±0.34 | <0.05 |
| Xu 2021 |  |  |  |  |  |  |  |  |
|  | ALT(U/L) | 54.68 ±25.03 | 58.76 ±38.24 | 0.696 | AST(U/L) | 41.10 ±14.84 | 43.42 ±23.57 | 0.684 |
|  | TBA(μmol/L) | 10.49 ±4.36 | 12.11 ±5.48 | 0.093 | Pruritus score | 0. 79 ± 0. 65 | 1. 06 ± 0. 60 | 0.041 |
| Zhu 2021 |  |  |  |  |  |  |  |  |
|  | ALT(U/L) | 10.73±1.52 | 24.89±4.86 | <0.05 | AST(U/L) | 6.52±1.37 | 12.43±2.01 | <0.05 |
|  | TBA(μmol/L) | 10.23±1.71 | 16.31±2.48 | <0.05 |  |  |  |  |

**Abbreviations:**

ALT, alanine aminotransferase; ALP, alkaline phosphatase; AST, aspartate aminotransferase; APOA1, Apolipoprotein A1; DBIL, direct bilirubin; CG, courage acid; GPx, glutathione peroxidase; GSH, Reduced glutathione; γGT, γ-glutamyl transpeptidase; ICP, Intrahepatic cholestasis of pregnancy; IL-17, interleukin 17; LPO, lipid peroxide; MDA, malondialdehyde; PPC, Polyene phosphatidylcholine; SAMe; S-adenosylmethionine; SOD, superoxide dismutase; TBIL, total bilirubin; TGF-β, transforming growth factor-β.

Data could not be exttracted: Frezza 1984, Frezza 1990 and Ribalta 1991.
